# Mixed effect estimation in deep compartment models: Variational methods outperform first-order approximations

**DOI:** 10.1007/s10928-024-09931-w

**Published:** 2024-07-04

**Authors:** Alexander Janssen, Frank C. Bennis, Marjon H. Cnossen, Ron A. A. Mathôt, for the OPTI-CLOT study group SYMPHONY consortium, S. H. Reitsma, F. W. G. Leebeek, M. Coppens, K. Fijnvandraat, K. Meijer, S. E. M. Schols, H. C. J. Eikenboom, R. E. G. Schutgens, F. Heubel-Moenen, L. Nieuwenhuizen, P. Ypma, M. H. E. Driessens, I. van Vliet, M. J. H. A. Kruip, S. Polinder, P. Brons, F. J. M. van der Meer, K. Fischer, K. van Galen, P. W. Collins, M. Mathias, P. Chowdary, D. Keeling, J. Lock, H. C. A. M. Hazendonk, T. Preijers, N. C. B. de Jager, L. Schutte, L. H. Bukkems, M. C. H. J. Goedhart, J. M. Heijdra, L. Romano, W. Al Arashi, M. E. Cloesmeijer, S. F. Koopman, C. Mussert

**Affiliations:** 1 Department of Clinical Pharmacology, Hospital Pharmacy, Amsterdam UMC, University of Amsterdam, Amsterdam, The Netherlands; 2 Emma Children’s Hospital, Amsterdam UMC, University of Amsterdam, Amsterdam, The Netherlands; 3 Department of Pediatric Hematology, Erasmus MC Sophia Children’s Hospital, Erasmus University Medical Center, Rotterdam, The Netherlands

**Keywords:** Machine Learning, Pharmacometrics, Pharmacokinetics, Estimation methods, Variational Inference

## Abstract

This work focusses on extending the deep compartment model (DCM) framework to the estimation of mixed-effects. By introducing random effects, model predictions can be personalized based on drug measurements, enabling the testing of different treatment schedules on an individual basis. The performance of classical first-order (FO and FOCE) and machine learning based variational inference (VI) algorithms were compared in a simulation study. In VI, posterior distributions of the random variables are approximated using variational distributions whose parameters can be directly optimized. We found that variational approximations estimated using the path derivative gradient estimator version of VI were highly accurate. Models fit on the simulated data set using the FO and VI objective functions gave similar results, with accurate predictions of both the population parameters and covariate effects. Contrastingly, models fit using FOCE depicted erratic behaviour during optimization, and resulting parameter estimates were inaccurate. Finally, we compared the performance of the methods on two real-world data sets of haemophilia A patients who received standard half-life factor VIII concentrates during prophylactic and perioperative settings. Again, models fit using FO and VI depicted similar results, although some models fit using FO presented divergent results. Again, models fit using FOCE were unstable. In conclusion, we show that mixed-effects estimation using the DCM is feasible. VI performs conditional estimation, which might lead to more accurate results in more complex models compared to the FO method.

**Supplementary Information:**

The online version contains supplementary material available at 10.1007/s10928-024-09931-w.

## Introduction

Non-linear mixed effect (NLME) models serve as the established methodology for the analysis of time-series data within the domain of pharmacometrics. These models allow for the simultaneous estimation of population and individual level effects using (semi-)mechanistic models, and are particularly useful for disentangling different sources of variability from data. The inclusion of random variables *η* imposes a distribution over the model parameters and can be thought of as representing the effect of unseen covariates. At prediction-time, an individual estimate of the parameters can be obtained based on the observations. Aside from improving prediction accuracy, these individual estimates can also be used to simulate drug exposure or effects based on unseen treatment strategies, facilitating the selection of optimal treatment on a personalized basis.

Recently, the field of pharmacometrics has seen an influx of interest in the use of machine learning (ML) methods [1–3]. Most ML techniques favour data-driven learning of relationships between covariates and observations based on large amounts of data. However, the availability of large data sets is often a limiting factor within the context of pharmacometrics, rendering most standard ML methods ineffective. Moreover, algorithms such as neural networks and tree-based methods require the utilization of drug dose as model input, which has been shown to be problematic for reliable extrapolation to unseen data [1, 4]. Combining prior knowledge with machine learning methods in so-called hybrid model architectures poses a promising alternative, potentially improving both data efficiency and predictive performance.

One such architecture is the deep compartment model (DCM), which uses neural networks to learn the relationship between covariates and the parameters of a system of differential equations representing the (semi-)mechanistic model [5]. This architecture is highly flexible: it supports all problems involving ODEs, can learn the effects of specific covariates only (using explicit equations for others), or can be used to learn the partial differential equations describing drug kinetics/dynamics or parts thereof using Neural-ODEs [4, 6, 7]. In its current form, the framework focusses on the estimation of fixed effects. As these models use highly flexible neural networks, failing to assign part of the variability to random effects can potentially result in the model internalizing noise. Another downside is that model predictions cannot be individualized, limiting its potential for use in clinical practice.

In the work by Lu et al., a variational auto-encoder (VAE) [8] is used to produce individual prior distributions over the Neural-ODE parameters, enabling the personalization of predictions [4]. In VAEs, neural networks are used to estimate parameters (e.g. mean and variances) for a set of random variables describing the Neural-ODE parameters. Optimization is simplified by amortization of the learning procedure [8, 9], often optimizing the mean squared error of predictions combined with a regularizing term restricting complexity of the latent variables (e.g. using hyper-priors such as a standard Normal). However, this approach breaks the typical assumption that random effects are independent of the covariates, and in practice often results in the variance of (part of) the latent variables shrinking to zero to benefit prediction accuracy [10, 11]. To circumvent these issues, estimation of random effects should be decoupled from the estimation of fixed-effects as is the case in classical NLME models.

The aim of this work is to formulate a robust approach to jointly estimate fixed and random effects within the DCM framework. We investigate the performance of classical first-order approximation methods used in NLME models as well as machine learning based variational methods [12]. The accuracy and stability of these different algorithms are tested on a simulated data set using a population pharmacokinetic (PK) approach. Finally, we showcase the use of the mixed-effect DCM on two real world data sets of haemophilia A patients receiving standard half-life (SHL) factor VIII (FVIII) concentrates during prophylaxis and surgery.

### Estimation of random variables

Given a data set of covariates ***X***, interventions ***I*** (e.g. drug administration), and measurements ***y*** for each subject *i* ∈ {1, …, *n*}, we typically use an ODE-based model *A(t)* to represent the evolution of *y*_*i*_ over time:

1 $${y}_{i}\left(t\right)=A\left(t;{\zeta }_{i},{I}_{i}\right)+\epsilon , \text{where}\; \epsilon \sim N\left(0,\Sigma \right)$$

Here, matrix ***I***_*i*_ contains individual treatment information with corresponding time points and *ζ*_i_ = *f*(*x*_i_; *θ*) are typical ODE parameters (e.g. PK parameters) whose relationship to the covariates *X* are described by a set of functions *f* with fixed effect parameters *θ*. Mixed effects models introduce a subject-specific random variable *η*_i_ ∈ ℝ^K^ on (part of) the parameters of the ODE in order to account for additional heterogeneity between subjects:

2 $${z}_{i}=g\left({\zeta }_{i},{\eta }_{i}\right), \text{where}\; {\eta }_{i}\sim N\left(0,\Omega \right)$$

Here, *z*_*i*_ represents the individual estimate of the ODE parameters and Ω is a *K* × *K* covariance matrix. We drop the subscript *i* in subsequent equations to reduce cluttering. Following from the Bayes rule $p\left(\eta |y\right)=p\left(y | \eta \right)p\left(\eta \right)/p\left(y\right)$, we can obtain maximum a posteriori (MAP) estimates of *η* based on the measurements *y* by maximizing the joint likelihood $p\left(y,\eta \right)=p\left(y | \eta\right)p\left(\eta \right)$. However, obtaining maximum likelihood estimates of the fixed effect parameters is more complicated. One way is to marginalize out the random variables, which results in a complex integral often lacking a closed-form solution:

3 $$p\left(y;\Theta \right)=\int p\left(y,\eta ;\Theta \right)d\eta , \text{where}\;\Theta =\left\{\theta ,\Omega ,\Sigma \right\}$$

Classical methods approximate this integral using a Laplace approximation around the mode of the random effects and linearize the model by performing a first-order Taylor expansion. This results in a Gaussian approximation of the random effect posterior, and is known as the First-Order Conditional Estimation (FOCE) extended least squares objective function (see supplementary data 1 for derivation) [13, 14]. When using the FOCE objective, the model iterates through producing MAP estimates of *η* followed by optimization of *Θ* based on the linearized model. Further approximation of the FOCE objective results in the FO objective function, where the mode of *η* is fixed at the population mean (i.e. zero), removing the need for the calculation of MAP estimates (see supplementary data 1) [15]. However, individual random effects are rarely located at zero (unless shrinkage is high) and the resulting objective function is less accurate. In practice, the FO method is only appropriate when the inter-individual variances are small [16].

### Variational inference

Model performance likely depends on the accuracy of the approximation. The Laplace approximation (and the FO and FOCE by extension) suffers especially when *η* posteriors are non-Gaussian, or have multiple modes. Alternatively, we can apply Markov Chain Monte Carlo (MCMC) methods to obtain samples of model parameters that converge to their true posterior distributions. Unfortunately, MCMC quickly becomes computationally prohibitive when the number of subjects and dimension of the random variables increases. This is especially the case when the fixed effects model is a neural network with ill-defined posterior distributions over its weights [17]. Fortunately, several approximate methods for Bayesian inference have been developed to reduce computational complexity.

A notable example is Variational Inference (VI), where the true posterior is approximated by a (simpler) variational distribution *q* [12]. The variational approximation is optimized by minimizing its Kullback–Leibler (KL) divergence with respect to the true posterior. Since the true posterior is unknown, the evidence lower bound (ELBO) is maximized instead, which places a lower bound on the marginal likelihood *p*(y) (see supplementary data 1):

4 $$\text{log}\;p\left(y\right)=\underbrace{{\mathbb{E}}_{q_{\phi} \left(\eta \right)}\left[\text{log}\;p\left(y,\eta \right) - \text{log}\;{q}_{\phi }\left(\eta \right)\right]}_{\text{ELBO}}+\underbrace{\text{KL}\left({q}_{\phi }\left(\eta \right)\Vert p\left(\eta | y \right)\right)}_{\text{divergence}}$$

Here, *q*_*φ*_ is a tractable distribution parametrized by *φ* (e.g. *φ* = {*μ, σ*} in the case of a Normal distribution). Since *p*(y) is a constant, maximizing the ELBO implicitly minimizes the KL divergence. An unbiased estimate of the expectation in Eq. 4 can be obtained using Monte Carlo methods, but the resulting gradients have high variance. Roeder et al. describe the path-derivative gradient estimator of the ELBO, which has the property that the gradient variance shrinks to zero as *q*_*φ*_(*η*) approaches p(*η | y)* [18]. This means that a potentially very close approximation of the true posterior can be obtained based on the chosen complexity of *q*_*φ*_. Choosing a Gaussian approximation will result in a similar approximation of the integral in Eq. 3 as with FOCE, albeit a stochastic one due to the Monte Carlo approximation in Eq. 4.

It is of interest to compare VI to the classical first-order approximations when using the DCM framework to see if there are differences in performance. Since VI performs conditional estimation, we expect improved performance over the FO method in more complex models. A potential benefit of VI over FOCE might be reduced computational time as MAP optimization over *η* is not required. It is also unknown how well these models will behave when simultaneously learning fixed and random effect parameters when covariate effects are learned during the optimization, as is the case in the DCM.

## Methods

### Synthetic data generation

A total of 500 samples of patient age, height, weight, blood group, and von Willebrand factor antigen (VWF:Ag) levels were simulated from a recently proposed generative model for haemophilia A patients [19]. This generative model implements non-linear relationships to represent the joint distribution over these covariates. Covariate relationships were based on a directed acyclic graph (DAG) representing the causal effects of the covariates. The resulting samples are more realistic than samples from multivariate normal or marginal distributions. After generating synthetic covariate data, factor VIII levels were simulated based on a hypothetical population PK model implementing the following covariate effects:

5 $$\begin{array}{l}CL=0.1\cdot\frac{\mathrm{weight}}{70}^{0.75}\cdot\left(\frac{\mathrm{leaky}\_\mathrm{softplus}(\mathrm{VWF}+100)}{55}+0.9\right)\\V_1=2.0\cdot\frac{\mathrm{weight}}{70}\cdot\exp(\eta_{2})\\Q=0.15\\ V_{2}=0.75\end{array}$$

where $$\mathrm{leaky}\_ \mathrm{softplus} \left(x, \alpha = \frac{1}{20}, \beta= \frac{1}{10}\right)= \alpha \cdot x + (1- \alpha) \cdot \frac{log (\mathrm{exp}(x \cdot \beta)+1)}{\beta}$$.

Each virtual patient was given a single dose of 25 IU/kg rounded to the nearest 250 IU. Random samples $\eta \sim N\left(0,\Omega \right)$ with $$\Omega =\left[\begin{array}{c}\begin{array}{cc}0.037& 0.0113\end{array}\\ \begin{array}{cc}0.0113& 0.017\end{array}\end{array}\right]$$ were drawn to produce individual estimates of the PK parameters. Next, simulated FVIII concentration–time curves were generated based on a two compartment model. FVIII measurements were collected at 4, 24, and 48 h after dose.

### Evaluating the accuracy of variational approximations

The accuracy of variational posterior approximations was determined by comparing learned random effect posteriors obtained from VI to those obtained from MCMC sampling when using the true model from the simulation. Posteriors were compared in two settings: (1) using the true typical PK and population parameters (i.e. Ω and Σ), and (2) when only using the true typical PK parameters (also approximating the posterior over Ω and Σ). Covariance matrices *M* were decomposed in terms of marginal standard deviations *S* and correlation matrix *C* such that *M* = *S · C · S’*. More information on prior and hyper-prior selection for the MCMC model can be found in supplementary data 2.5.

For the MCMC model in scenario 1, a single chain was run to generate 10000 posterior samples using the NUTS algorithm. In scenario 2, 5000 samples were taken. Models were fit to the first data fold of the simulated data set, and 20 replicates of the VI algorithm were fit to compare to results from MCMC. The same prior distributions were used in the VI model. Posterior similarity was determined based on visualizations and quantified using the Wasserstein distance. The ADAM optimizer using a learning rate of 0.1 was used.

### Comparison of methods for estimating random variables

Given our computational budget, we decided on fitting 100 models for each of the methods. The complete data set was divided into 20 random subsets of 60 subjects drawn *with replacement* for model training with the remaining samples for determining model accuracy. Previous results indicated that data from 60 subjects was sufficient to fit accurate models [5, 20]. On each data fold, five replicates of model training were performed which we deemed to be a minimal requirement to represent variability induced by random initialization of model parameters. We chose to run a larger number of training replicates over data folds rather than within a single data fold (i.e. 20 vs. 5) as we assumed that the specific training data had a larger effect on parameter variability compared to random initialization following previous findings [21].

A multi-branch network based architecture of the DCM [21] was fit to each training fold of the simulated data set. In a multi-branch network, covariates are linked to specific ODE parameters such that each covariate effect is learnt in isolation. This contrasts standard fully-connected networks where all covariates are linked to all ODE parameters, potentially making the model susceptible to learning spurious covariate effects. In addition, the approach enables the direct visualization of learned functions for each of the covariates, making the model inherently interpretable without the need for post-hoc ML explanation methods. Subject weight and VWF:Ag were used as covariates. Global parameters were estimated for *Q* and *V*_*2*_. In the multi-branch network, weight was connected to *CL* and *V*_*1*_, and VWF:Ag was connected to *CL*. The same model was optimized using each of the objective functions. For each training replicate, random initial parameters were drawn from initial distributions. More information on model architecture and initial parameter settings can be found in supplementary data 2.

Again, covariance matrices M were decomposed in marginal standard deviations and correlation matrices. All variance estimates were constrained to be positive using the softplus function. Models were compared based on the root mean squared error (RMSE) of typical predictions, accuracy of the estimated population parameters (represented by the KL divergence of *Ω* and mean absolute error (MAE) of *σ*), and the similarity of the learned functions with respect to the true covariate effects. Models were fit based on the MSE (no estimation of population parameters), FO, FOCE, and VI objective functions. When using the VI objective, random effect posteriors were approximated using full-rank multivariate normal distributions. The expectation in the ELBO was approximated using Monte Carlo simulation, taking three random samples and using the reparametrization trick [8] to generate samples from *q*. For the models trained using FOCE, MAP estimates of the random effects were obtained by minimization of the negative joint likelihood for each subject using the BFGS method at the start of each epoch of training. Estimates were constrained between [-3, 3] to improve stability during optimization.

**Fig. 1:**
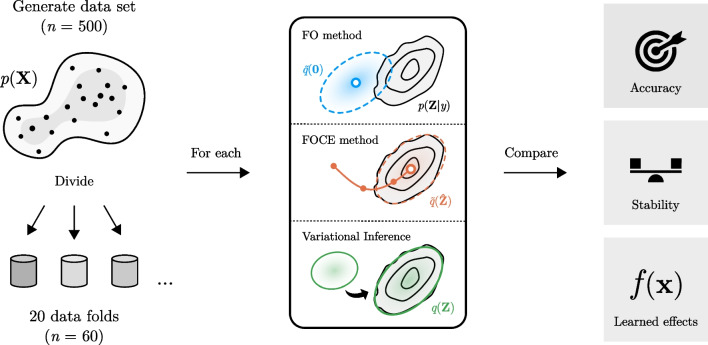
Comparison of the different methods in the simulation study

Models were trained for 2000 epochs and parameters were saved every 25 epochs to determine model convergence and stability during training. Most models converged within 250 – 500 epochs, so additional training iterations allowed insights into parameter stability after convergence and risks of overfitting when overextending training time. The ADAM optimizer using a learning rate of 0.1 or 0.01 was used depending on training stability. Results at the end of optimization were compared based on the mean of saved parameter estimates from the last 500 epochs of training. Uncertainty estimates over model parameters were obtained by taking the standard deviation of final parameter estimates for each of the training replicates.An overview of the approach is shown in Fig. 1.

First, a data set was simulated containing 500 virtual subjects based on a previously published generative model *p*(**X**). The data set was divided in 20 random data subsets with replacement to create the training (*n* = 60) and testing (*n* ≈ 440) data sets. On each data fold, models were fit using based on the different methods (FO, FOCE, and VI). In the FOCE method, a Gaussian approximation $\widetilde{q}$ of the random effect posterior *p*(**Z**|*y*) centered at its maximum a posteriori estimate (white circle) is obtained. In the FO method, the mode is fixed at zero, resulting in lower accuracy due to a potential mismatch with the true posterior. In VI, the divergence between a variational approximation *q*(**Z**) and the true posterior is minimized. After fitting the models, the methods were compared based on the accuracy of parameter estimates, their stability during training, and the similarity of learned covariate effects to true effects.

### Evaluation on real world data

The performance of the algorithms was also evaluated on two real world data sets of haemophilia A patients receiving SHL FVIII concentrates during prophylaxis (data set one) and following surgery (data set two). The data originates from the OPTI-CLOT clinical trial [20], were FVIII consumption was compared between standard weight-based dosing regiments and PK-guided dosing in moderate and severe haemophilia A patients undergoing surgery. The first data set contains a total of 69 subjects who received a PK profile following a 25–50 IU/kg test dose of one of five SHL FVIII concentrates. Three FVIII measurements were collected roughly 4, 24, and 48 h after administration. Available covariates were haemophilia severity, body weight, height, age, and VWF:Ag levels. A large proportion of VWF:Ag levels were missing (65.2%), with some subjects missing body weight or height data (1.4% and 4.3%, respectively). Missing values were imputed based on the mode of prior distributions produced by the generative model (i.e. the same model used for generation of the synthetic data) [19].

The second data set contained data on 66 subjects from data set one who underwent a minor or moderate risk surgical procedure within 12 months after their PK assessment. FVIII levels were measured before and after surgery and FVIII peak and trough levels were collected during follow-up. Compared to the first data set, follow-up time was longer (median of 144 vs 44 h) and subjects received a more complex combination of bolus doses and continuous infusions. Available covariates were haemophilia severity, body weight, height, VWF:Ag and VWF activity (VWF:act) levels, pre-assessed surgical risk scores, blood loss, and NaCL administration during surgery. In this data set, most subjects had multiple VWF measurements. Missing VWF:Ag values were imputed based on the mode of the prior distributions from the generative model multiplied by a factor of 1.3 (VWF:Ag levels are higher following surgery [22]). This factor was calculated from the mean difference between imputed VWF levels in data set one and average VWF levels per subject in data set two. The mean VWF:Ag value was used for each individual.

We fitted a multi-branch DCM with either an additive or combined residual error model to both data sets. Subject *CL* and *V*_*1*_ was predicted based on fat-free mass (FFM) calculated from body weight, BMI, and age using Al Sallami’s equation [23], with an additional effect of VWF:Ag on *CL*. Random effects were estimated for *CL* and *V*_*1*_ and global parameters were estimated for *Q* and *V*_*2*_. These choices match the results from a recent study on the PK of FVIII [19]. The goal of our analysis was to compare results from the different algorithms rather than to produce optimal models for these two data sets. For this reason, no additional covariate selection was performed. Models were trained until convergence (roughly 1000 epochs for MSE, FO, and VI; 2000 for FOCE) and parameters were saved every 25 epochs. Mean parameters from the last 250 epochs were presented. The ADAM optimizer with a learning rate of 0.1 was used. A larger number of epochs (2000 instead of 1000) were required for the FOCE model to converge when using a lower learning rate (0.01 instead of 0.1). Models were again compared based on the accuracy of typical predictions, final parameter estimates and their stability during training, and the learned functions.

### Model code

Model code and the simulated data set are available at https://github.com/Janssena/ME-DCM.jl.

## Results

### Accuracy of variational approximations compared to MCMC

**Fig. 2:**
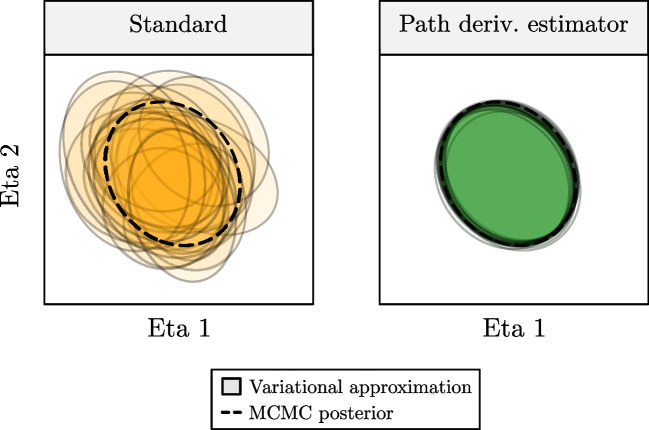
Accuracy of variational approximations of the random effect posterior obtained trough MCMC

First, we compared the accuracy of the variational posterior approximations obtained using VI to those obtained from MCMC. In Fig. 2, we can see that applying the path derivative gradient estimator results in accurate posteriors approximations and low variability across replicates compared to the standard estimator. Results for the two scenarios (with and without estimation of Ω and Σ posteriors) are summarized in supplementary Table 1. Approximate posteriors were most similar (represented by the Wasserstein distance) to the MCMC posteriors when using the path derivative gradient estimator. In both scenarios, variational posteriors of the individual random effects were highly accurate (see supplementary Fig. 1). Contrastingly, posteriors for the population parameters were less accurate as variational posteriors tended to underestimate the variance of the MCMC posteriors. We focus the remainder of the manuscript on results obtained using the path derivative estimator.

95% confidence regions of the posterior produced by MCMC (dashed lines) and VI (coloured ellipses) are shown for a single subject across 20 replicates of model training. Variational approximations when using the standard VI algorithm (left figure) and the path derivative estimator (right figure) are shown. The path-derivative estimator results in highly accurate posterior approximations compared to the standard VI objective.

### Comparison of VI to first-order objectives

Next, we compare the performance of the different objective functions on the simulated data. We found that models fit using the FOCE objective function behaved erratically during optimization. Several models failed optimization (non-positive definite Ω) which seemed to be related to the specific formulation of the objective function used (supplementary Fig. 2). A reduction of the learning rate (from 0.1 to 0,01) also improved stability of models fit using FOCE (data not shown). In the remainder of the manuscript we thus show results from the FOCE formulation based on Eq. s10 using a learning rate of 0.01 (supplementary data 1).

**Fig. 3:**
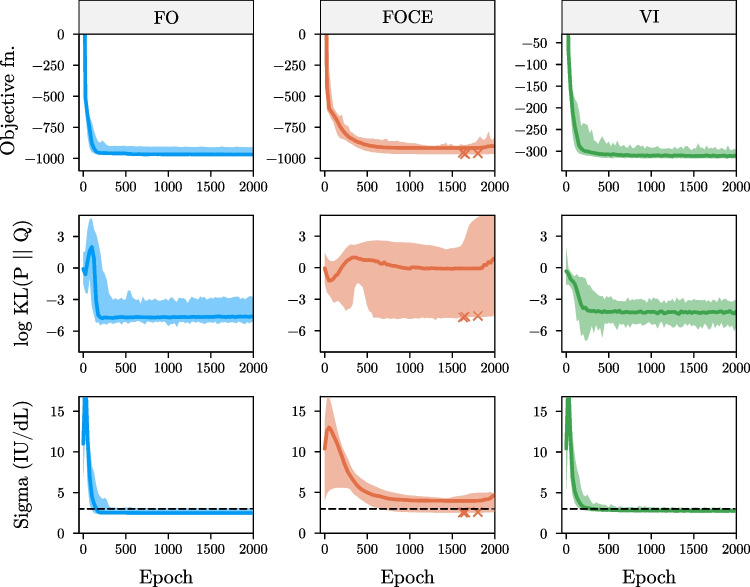
Objective function value and parameter accuracy during training on the simulated data

In Fig. 3, we display the objective function value, log KL divergence of Ω, and residual error estimate during training for the FO, FOCE (Eq. s10 + reduced learning rate), and VI objectives. We notice that the FO and VI objectives quickly converge to accurate estimates of the population parameters. These models were not affected by an over-extension of training time, as judged by the stability of parameter estimates during the final 1500 epochs. In contrast, large fluctuations in the KL divergence of Ω are observed when using the FOCE objective. These fluctuations are not always reflected by the objective function value, making it difficult to determine actual model convergence. Looking at the individual elements of the Ω matrix (i.e. marginal standard deviations S and correlation matrix C), we notice that estimates obtained using FOCE generally underestimated the variances (supplementary Fig. 2).

Objective function value (top row), log KL divergence of Ω (middle row), and the residual error estimate (bottom row) are shown for the models fit using the FO, FOCE, and VI method. Solid lines indicate median value across replicates along with 95% confidence intervals. Dashed line indicates the true value of the additive error (sigma). Crosses indicate models that failed optimization. Models fit using the FOCE objective present higher bias of estimated and lower stability during training.

The results at the end of optimization for the MSE, FO, FOCE, and VI objectives are summarized in Table 1. All methods resulted in similar median root mean squared error of typical predictions. Results for the FO and VI objectives were highly similar, with low error of population parameter predictions. Models fit using the FOCE objective displayed biased parameter estimates as well as high variability between replicates. We can see that models fit using VI completed training slightly faster than models fit using FO (median run time of 14.7 vs. 16.2 min), with FOCE models taking significantly longer (37.7 min). The computational burden of VI can potentially be further reduced close to the training time of MSE-based models by decreasing the number of Monte Carlo samples to 1 (median run time of 5.2 min) without loss of parameter accuracy (see supplementary Table 2).

**Table 1:** Accuracy of model parameters after convergence for the simulated data set

Method	Run time (minutes; median ± SD)	RMSE (IU/dL; median ± SD)	KL divergence of Ω (median ± SD)	MAE of ω_1_ ± SD	MAE of ω_2_ ± SD	MAE of additive error ± SD (IU/dL)
MSE	3.2 ± 0.73	6.34 ± 0.37	-	-	-	-
FO	16.2 ± 6.5	5.86 ± 0.25	0.009 ± 0.01	0.011 ± 0.01	0.0087 ± 0.001	0.47 ± 0.12
FOCE (Eq. s10)	37.7 ± 7.5	5.75 ± 0.36	1.0 ± 313	0.11 ± 0.05	0.046 ± 0.03	0.92 ± 0.60
VI	14.7 ± 2.6	5.80 ± 0.59	0.011 ± 0.005	0.013 ± 0.008	0.0086 ± 0.002	0.23 ± 0.03

*SD* standard deviation, *RMSE* root mean squared error, *KL* Kullback–Leibler, *MAE* mean absolute error

Results for the models at the end of convergence are shown. Parameter estimates obtained from the FOCE objective function presented higher error and variability between training replicates.

Finally, we investigate the learned functions at the end of optimization for each of the models (supplementary Fig. 4). For all objectives, median covariate effects were very similar to the ground truth functions used in the simulation. Interestingly, we notice a low degree of bias of the learned covariate effects when using the FOCE objective, even though the population parameters were inaccurate. Compared to the mixed-effects models, use of the MSE objective seemed to potentially result in a higher degree of variance in the learned effects between model replicates.

### Comparison on real world data

Next, we evaluated the performance of the different algorithms on two real-world data sets. Patient characteristics for both data sets are shown in Table 2. Models fit using a combined error model depicted at least a 20 point decrease in objective function value for all methods. In Table 3, we show the final parameter estimates for the models with combined error. Models fit using FO or VI resulted in similar median parameters estimates after convergence. However, parameter estimates in some of the replicates of the FO model were less stable, most notably with respect to *ω*_*1*_ and the proportional error estimate (see supplementary Fig. 5). Parameter estimates obtained from the FOCE method were again different from the other algorithms. Both the *ω*_*2*_ and additive error estimates were notably higher in both data sets. Again, the FOCE objective function value was a poor indicator of model convergence, with parameters still changing after apparent convergence (see supplementary Fig. 5). In contrast, models fit using VI quickly converged and parameter estimates were stable.

**Table 2:** Patient characteristics for the two real-world data sets

Covariate	Data set one: PK profiles (n = 69)		Data set two: following surgery (n = 66)	
	Number (%-age) or mean [range]	Number of entries with missing values (%)	Number (%-age) or mean [range]	Number of entries with missing values (%)
Body weight (kg)	86.0 [50.4—134]	1 (1.4%)	85.7 [50.4—134]	0 (0%)
Height (cm)	179 [148—198]	3 (4.3%)	178 [148—198]	0 (0%)
Age (years)	47.6 [12.1—76.9]	0 (0%)	47.6 [12.4—76.9]	0 (0%)
Blood group		0 (0%)		0 (0%)
- A	19 (28%)		18 (27%)	
- B	3 (4.3%)		3 (4.5%)	
- AB	5 (7.2%)		5 (7.5%)	
- O	42 (61%)		40 (61%)	
Pre-assessed surgical risk		NA		0 (0%)
- Low	NA		35 (53%)	
- Medium	NA		31 (47%)	
Haemophilia severity		0 (0%)		0 (0%)
- Moderate	22 (32%)		22 (33%)	
- Severe	47 (68%)		44 (67%)	
Expected blood loss		NA		0 (0%)
- Mild	NA		42 (64%)	
- Moderate	NA		24 (36%)	
Blood loss during surgery (mL)	NA	NA	227 [0—1200]	21 (32%)
Brand of FVIII concentrate		0 (0%)		0 (0%)
- Octocog alfa (Kogenate^©^)	18 (26%)		18 (27%)	
- Octocog alfa (Advate^©^)	22 (32%)		21 (32%)	
- Moroctocog alfa (ReFacto AF^©^)	4 (5.8%)		4 (6.1%)	
- Plasma-derived FVIII Concentrate (Aafact^©^)	3 (4.3%)		3 (4.5%)	
- Turoctocog alfa (NovoEight^©^)	22 (32%)		20 (30%)	
VWF:Ag (%)	113 [61—225]	45 (65.2%)	131 [0.43—384]	9 (13.6%)
VWF:act (%)	106 [58—185]	45 (65.2%)	127 [32—396]	9 (13.6%)
FVIII measurements per patient	3.26 [3–10]	-	8.61 [2–21]	-

*kg* kilogram, *cm* centimeter, *FVIII* blood clotting factor VIII, *aPTT* activated partial thromboplastin time, *s* seconds, *PT* Prothrombin time, *VWF* von Willebrand factor, *NA* not applicable

**Table 3:** Accuracy of model parameters on real world data sets

Method	Run time (minutes; median ± SD)	RMSE (IU/dL; median ± SD)	Median ω_1_ (%CV) ± SD	Median ω_2_ (%CV) ± SD	Median additive error (IU/dL) ± SD	Median proportional error ± SD
Data set one (prophylactic setting)						
MSE	2.1 ± 0.16	14.1 ± 0.24	-	-	-	-
FO	9.1 ± 2.2	14.3 ± 0.77	0.289 (29.5) ± 0.044	0.127 (12.8) ± 0.020	3.09 ± 0.43	0.105 ± 0.013
FOCE (Eq. s10)	54.2 ± 14^a^	19.0 ± 4.3	0.240 (24.4) ± 0.019	0.465 (49.1) ± 0.052	3.70 ± 0.05	0.108 ± 0.004
VI	8.0 ± 0.51	14.3 ± 0.69	0.282 (28.8) ± 0.012	0.160 (16.1) ± 0.004	2.89 ± 0.077	0.094 ± 0.017
Data set two (perioperative setting)						
MSE	2.3 ± 0.17	27.6 ± 1.13	-	-	-	-
FO	19.5 ± 3.8	32.0 ± 1.66	0.300 (30.7) ± 0.012	0.211 (21.3) ± 0.018	2.89 ± 1.63	0.151 ± 0.012
FOCE (Eq. s10)	113 ± 20^a^	31.5 ± 1.66	0.321 (32.9) ± 0.014	0.326 (33.5) ± 0.020	4.53 ± 0.37	0.152 ± 0.005
VI	14.6 ± 1.2^b^	30.0 ± 1.17	0.316 (32.4) ± 0.005	0.179 (18.0) ± 0.001	2.46 ± 0.024	0.165 ± 0.001

*SD* standard deviation, *RMSE* root mean squared error, *CV* coefficient of variation

^a^ = convergence after 2000 epochs, ^b^ = convergence after 1250 epochs,

Patient characteristics and missing data are shown for data set one and two. A point to note are the differences in the amount of missing data between the two clinical settings. Most prominently, VWF:Ag levels were missing for most (65%) subjects in data set one.

Coefficient of variation was calculated using the following formula: $CV\left(\%\right)=\sqrt{\text{exp}\left({\omega }^{2}\right)-1}\cdot 100\%$. Compared to the other methods, the FOCE objective results in divergent parameter estimates. Higher RMSE in data set two is indicative of the higher inter-individual variability in FVIII levels observed during surgical procedures.

**Fig. 4:**
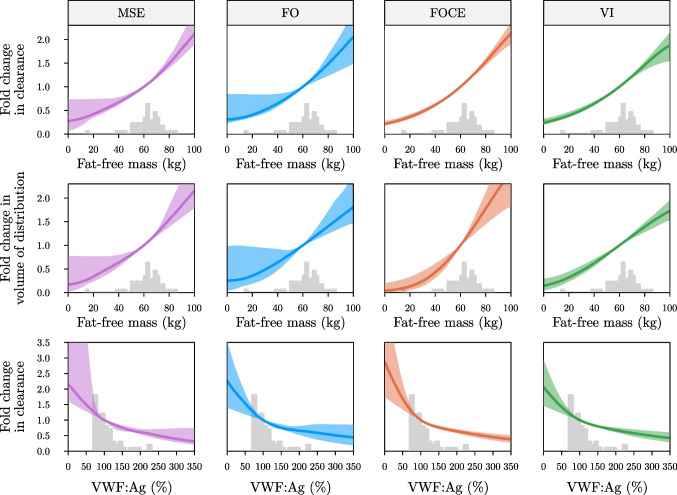
Learned covariate effects from models fit on real-world data set one

Visualization of covariate effects can help to provide insights in the covariate effects learned by the models, as well as regions of higher uncertainty due to data sparsity in parts of the covariate space (see Fig. 4). Learned functions in the perioperative setting (data set two) were similar to those learned based on the PK profiles (see Fig. 4 and supplementary Fig. 6). Lower uncertainty over the learned functions was observed when using FOCE, but this result could be replicated for the other objectives by lowering the learning rate (see supplementary Fig. 7).

Covariate effects for models fit using the MSE (left column), FO (centre left column), FOCE (centre right column), and VI (right column) are shown. Learned functions are shown for the effect of fat-free mass on clearance (top row), fat-free mass on volume of distribution (middle row) and von Willebrand factor antigen levels on clearance (bottom row) at the end of training on data set one. Median covariate effect (solid line) along with 95% confidence intervals are shown. Grey histograms represent the corresponding covariate distributions.

## Discussion

In this work, we investigated the performance of classical first-order approximations as well as ML-based variational methods for estimating mixed-effects in DCMs. Results from our simulation experiment suggest that both the FO and VI objectives reliably converged to accurate solutions, whereas the FOCE objective function resulted in biased estimates and high variability amongst training replicates. These results were replicated in two real-world data sets, where we again observed divergent results when using the FOCE objective. Here, VI resulted in the most reliable results as some models fit using FO depicted lower parameter stability during training. Learned covariate effects for all models could be visualized by using the multi-branch architecture of the DCM. This enables model interpretation and is useful for critiquing the model during development.

Even though the FOCE objective function is widely regarded to be more accurate than the FO method, our results indicate that this is not always the case. When the underlying model is highly flexible and is trained using gradient descent, as is the case when using neural networks, the FOCE algorithm seemed to result in poor convergence behaviour. Although a different formulation of the objective function and lowering of the learning rate slightly improved results, optimization still was not reliable. Population parameter estimates were highly variable during training, even after apparent convergence based on the stabilization of the objective function value. We hypothesize that frequent changes to the loss landscape affect the stability of optimization when using gradient descent. Since the fixed effects model initially has low accuracy, early *η* estimates shrink to the prior mean with relatively high posterior variance. As a result, the prior variances (*Ω*) might have a tendency to shrink to zero. After a few iterations, the accuracy of typical PK parameter improves, resulting in jumps in the estimates of *η* away from zero and potentially large changes to the loss landscape. Methods such as gradient descent might perform poorly in such settings, getting stuck in poor local optima and frequently changing the direction of gradients in response to changes to the loss landscape. For both the FO objective and VI such changes do not occur, since the random effects are either fixed during training (as in FO) or part of the parameter space (as in VI). Additional research is needed to investigate why the FOCE objective fails in this setting.

As an alternative to the FOCE objective, we suggest VI for the concurrent optimization of fixed effect parameters and subject-specific random effect posteriors. We show that variational posteriors were very accurate when using the path derivative gradient estimator, which is simple to implement. Most probabilistic programming languages such as Turing.jl or Pyro provide functionality for fast implementation of VI [24, 25]. Results from our experiments indicate fast and stable convergence to an accurate set of parameter estimates. Additional benefits of VI are improved computational speed compared to FOCE (even outperforming FO for one of our data sets) as well as it being part of an active field of research, potentially bringing more improvements in terms of speed and accuracy [26]. Furthermore, the complexity of the variational approximation can be controlled, making the method suitable for problems where the random effect posterior is multi-modal or better described by a more complex distribution by for example using Gaussian mixture models or normalizing flows based variational posteriors, respectively [18, 27]).

VI is conceptually very similar to (stochastic) expectation maximization (EM) procedures [28, 29]. In Stochastic approximation EM (SAEM), samples from the random effect posterior are taken (for example using MCMC) and a stochastic averaging procedure with adaptive step sizes is performed to approximate the integral in Eq. 3 [29]. This is followed by maximization of the fixed-effects parameters based on the obtained approximation. In VI, samples are instead taken from a Variational distribution whose parameters are directly optimized along with the fixed-effects parameters. A benefit of the latter approach is that we obtain a closed-form expression for the random effect posterior and that no adaptive step size procedures are required. It might be of interest to compare the performance of these two approaches to see if there are notable differences.

Even though the FO method resulted in reasonable median parameter estimates in our experiments, the use of VI might be preferred. In more complex models, FO is likely to result in less accurate parameter estimates. We already found that some training replicates on the second real-world data set showed signs of lower stability and poor accuracy. It has been shown that the FO method can often produce biased parameter estimates with incorrect uncertainty estimates in certain settings [30]. Furthermore, it is well known that the FO method is not suited for problems with high levels of inter-individual variability [16]. Especially in the context of pharmacodynamic (PD) models, this variability is expected to be relatively large (often > 100% coefficient of variation) and so the FO method might be unsuited in most cases. In contrast, accuracy of VI depends on the chosen variational approximation (Gaussian approximations are often sufficient) and the number of Monte Carlo samples, both of which can be adapted based on the complexity of the problem at hand.

There were also some limitations to this work. First, our results indicated that variational approximations estimated over population parameters depicted an underestimation of posterior variance compared to MCMC. Unfortunately, estimation of the population parameter posteriors using MCMC is computationally intensive as it still requires iteration over all subjects in the data set. This might only be feasible in small data sets (e.g. ≤ 30 subjects) and when using relatively simple models (simple ODEs, small neural network, and small number of random effect parameters). To estimate uncertainty over model parameters we might need to resort to deterministic methods to estimate standard errors. Similar to the approach used by NLME models, reasonable estimates can be obtained based on post-hoc Gaussian approximations based on the Fisher information matrix. Second, we use deterministic methods to optimize neural network weights. Since models could be prone to overfitting, we might want to marginalize over predictions from many model replicates to reduce spurious effects and to obtain estimates of functional uncertainty. Ideally, uncertainty over covariate effects can be estimated in a single model replicate. Alternatively, the use of priors over the desired function space in this context can be of interest in order to regularize function complexity. It would be of interest to investigate how these improvements can be implemented in practice. Finally, we did not perform an exhaustive evaluation of the performance of the objective functions in many different data sets, different degrees model complexity, or for very different initial parameter and prior distributions settings. More research might be desirable to evaluate the performance of VI in multiple practical settings.

## Conclusion

In summary, our work introduces mixed-effects estimation in the DCM framework. Highly accurate posterior approximations for the random effects could be obtained using VI, and estimated population parameters were accurate and stable during training. We found that the FOCE method did not provide reliable results and might not be suited for this purpose. In our experiments, VI was the most reliable approach for the estimation of mixed effects and might perform better in more complex models compared to FO. Mixed-effects models enable the individualization of predictions based on clinical measurements, enhancing the likelihood of the clinical adoption of these algorithms. This extension to the DCM framework further promotes the use of ML-based methods as a viable alternative to classical NLME models.

## Supplementary Information

Below is the link to the electronic supplementary material.

Supplementary file1 (PDF 8521 KB)

## Data Availability

No datasets were generated or analysed during the current study.
