# Supplementary material for: Mixed effect estimation in deep compartment models: Variational methods outperform first-order approximations
Source: J Pharmacokinet Pharmacodyn. 2024 Jul 4;51(6):797–808. doi: 10.1007/s10928-024-09931-w (PMC11579087; doi:10.1007/s10928-024-09931-w)
Supplement: Supplementary file 1 — Supplementary file1 (PDF 8521 KB) [file 10928_2024_9931_MOESM1_ESM.pdf]

# Supplementary data 1. Derivation of objective functions

## 1.1 Laplace approximation

Given a complex integral of the form $\int f(x)dx$, where $f(\cdot)$ is a twice-differentiable function, $f(x)$ can be re-expressed as $g(x) = \log f(x)$, such that $\int f(x)dx = \int \exp g(x)dx$. Consider the second-order Taylor series expansion of $g(x)$ around a point $x_z0$:

$$g(x) \approx g(x_0) + g'(x_0)(x - x_0) + \frac{1}{2}g''(x_0)(x - x_0)^2 \tag{s1}$$

If we set $x_0$ to be the mode of $g(x)$ the second term becomes zero (since $g'(x_0) = 0$). We thus obtain the following approximation of the integral:

$$\int f(x)dx \approx \int \exp(g(x_0) + \frac{1}{2}g''(x_0)(x - x_0)^2)dx \tag{s2}$$

Since $\exp g(x_0) = f(x_0)$ is a constant, we can move it out of the integral to obtain:

$$\begin{aligned} \int \exp(g(x_0) + \frac{1}{2}g''(x_0)(x - x_0)^2)dx &= f(x_0) \cdot \int \exp(\frac{1}{2}g''(x_0)(x - x_0)^2)dx \\ &= f(x_0) \cdot \sqrt{\frac{2\pi}{-g''(x_0)}} \end{aligned} \tag{s3}$$

The second term in the last equation originates from integration of the probability density function of a normal distribution:

$$\int p(X)dx = \frac{1}{\sigma\sqrt{2\pi}} \cdot \int \exp\left(-\frac{1}{2\sigma}(x - \mu)^2\right) dx = 1 \tag{s4}$$

From Eq. s4 we can see that $\sigma \cdot \sqrt{2\pi} = \int \exp\left(-\frac{1}{2\sigma}(x - \mu)^2\right)$. If we set $\sigma = -g''(x_0)^{-1}$ we recover the second term in Eq. s3. The Laplace approximation thus results in a Gaussian approximation around the model of the random effects:

$$\int f(x)dx \approx f(x_0) \cdot \sqrt{\frac{2\pi}{-g''(x_0)}} \tag{s5}$$

In the context of non-linear mixed effects models this results in the following objective function after simplification:

$$\mathcal{L}(\Theta, \hat{\eta}) = p(y \mid \hat{\eta}; \Theta) + \log|\Omega| + \hat{\eta} \cdot \Omega^{-1} \cdot \hat{\eta}^T + \left|\Omega^{-1} + \frac{H(\hat{\eta})}{2}\right| \tag{s6}$$

Where $H$ is the hessian of the likelihood with respect to $\eta$: $H(\eta) = \frac{\partial^2 p(y|\eta)}{\partial\eta}$.

## 1.2 First-order conditional estimation (FOCE)

To avoid the computation of the second order derivatives, the Hessian matrix can be approximated as a function of the Jacobian vector of $\hat{\eta}$:

$$\mathbb{E}\left[H(\eta)\right] \approx \frac{1}{2}\mathbb{E}\left[J(\eta) \cdot J(\eta)^T\right] \tag{s7}$$

This additional approximation results in the first-order conditional estimation objective function (also see [1]):

$$\mathcal{L}(\Theta, \hat{\eta}) = p(y \mid \hat{\eta}; \Theta) + \log|\Omega| + \hat{\eta} \cdot \Omega^{-1} \cdot \hat{\eta}^T + \left|\Omega^{-1} + \frac{\mathbb{E}\left[J(\eta) \cdot J(\eta)^T\right]}{4}\right| \tag{s8}$$

We can further simplify this equation to obtain the FOCE objective function that is used in NONMEM:

$$-2\mathcal{L}(\Theta, \hat{\eta}) = \log|C| + \frac{(y - A(t; \hat{z}, I) + J(\hat{\eta}) \cdot \hat{\eta})^2}{C} \tag{s9}$$

Where $C = J(\hat{\eta}) \cdot \Omega \cdot J(\hat{\eta})^T + \Sigma$ and $\hat{z}$ is the individual estimate of the ODE parameters based on $\hat{\eta}$. The jacobian is calculated with respect to the output of the ODE. AN equivalent expression exists:

$$-2\mathcal{L}(\Theta, \hat{\eta}) = \log|C| + \frac{(y - A(t; \hat{z}, I))^2}{\Sigma} + \hat{\eta} \cdot \Omega^{-1} \cdot \hat{\eta}^T \tag{s10}$$

We use this objective function (Eq. s10) in the manuscript.

## 1.3 The FO objective

In the FO objective, the mode of $\eta$ is assumed to be located at the population mean (i.e. zero). This results in the following objective function following from Eq. s9:

$$-2\mathcal{L}(\Theta, \hat{\eta}) = \log|C_0| + \frac{(y - A(t; z_0, I))^2}{C_0} \tag{s11}$$

where $C_0 = J(0) \cdot \Omega \cdot J(0)^T + \Sigma$.

## 1.4 Derivation of the ELBO

In Bayesian inference, given a set of observations $\mathbf{X} = \{\mathbf{x}_1, \ldots, \mathbf{x}_n\}$ we are often interested in obtaining the posterior distribution over a set of latent variables $\mathbf{Z} = \{\mathbf{z}_1, \ldots, \mathbf{z}_n\}$. We are however often unable to compute the model evidence $p(\mathbf{X})$ as this requires integration over all possible values of $\mathbf{Z}$. The goal of Variational Inference (VI) is to instead minimize the differences between the true posterior and a (simpler) variational approximation $q(\mathbf{Z})$. One way to represent the differences between two distributions is via their KL-divergence:

$$\begin{aligned}
\mathrm{KL}(q(\mathbf{Z}) \| p(\mathbf{Z} \mid \mathbf{X})) &= \int q(\mathbf{Z}) \log \frac{q(\mathbf{Z})}{p(\mathbf{Z} \mid \mathbf{X})} dz \\
&= \mathbb{E}_{q(\mathbf{Z})} \left[ \log \frac{q(\mathbf{Z})}{p(\mathbf{Z} \mid \mathbf{X})} \right] \\
&= \mathbb{E}_{q(\mathbf{Z})} \left[ \log q(\mathbf{Z}) \right] - \mathbb{E}_{q(\mathbf{Z})} \left[ \log p(\mathbf{X}, \mathbf{Z}) \right] + \log p(\mathbf{X}) \qquad \text{(s12)}
\end{aligned}$$

We can rewrite this expression to obtain:

$$\log p(\mathbf{X}) = \underbrace{\mathbb{E}_{q(\mathbf{Z})} \left[ \log p(\mathbf{X}, \mathbf{Z}) - \log q(\mathbf{Z}) \right]}_{\text{ELBO}} + \underbrace{\mathrm{KL}(q(\mathbf{Z}) \| p(\mathbf{Z} \mid \mathbf{X}))}_{\text{divergence}} \qquad \text{(s13)}$$

Note that the KL divergence is an asymmetric measure, i.e. $\mathrm{KL}(q(\mathbf{Z}) \| p(\mathbf{Z} \mid \mathbf{X})) \neq \mathrm{KL}(p(\mathbf{Z} \mid \mathbf{X}) \| q(\mathbf{Z}))$. Swapping terms in the KL divergence results in a different objective function with different behaviour.

# Supplementary data 2. Model architecture

## 2.1 Multi-branch network

In a multi-branch neural network architecture, the covariates are connected to independent sub-networks, such that the model learns the effect of each covariate in isolation. The independent covariates are combined using a product, similar to the common implementation of covariates in non-linear mixed effects models. In this sense, the architecture is similar to a generalized additive model, using product accumulation rather than the sum of covariate effects. Typical fully-connected neural networks can learn complex interactions between the covariates. By removing the possibility of learning such potentially spurious correlations, model performance and generalizability can potentially be improved. Similarly, we can specifically link covariates with known causal effects on one of the parameters, preventing the model from learning any spurious effects with respect to the other parameters. An additional benefit of the approach is that the output of each sub-model can be visualized, allowing for the interpretation of the learned covariate effects. A schematic representation of fully-connected and multi-branch networks is provided below.

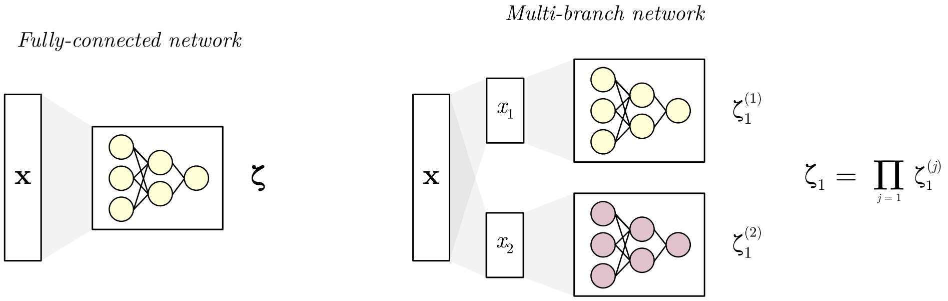


## 2.2 Model architecture

A multi-branch architecture was used to learn the effect of weight (or fat-free mass in the real-world experiment) on clearance and volume of distribution, and the effect of von Willebrand factor antigen (VWF:Ag) levels on clearance. The first model consisted of a single hidden layer containing 12 neurons feeding into a transformed softplus activation function: $\pi(x) = \frac{1}{10} \cdot \log(\exp(10 \cdot x) + 1)$. Inputs were normalized between roughly 0 and 1 by dividing model input by 150 kg. Output from this hidden layer was fed into two independent hidden layers, each again consisting of 12 neurons connected to a single output neuron. The two independent output neurons represent the effect of the covariate on clearance or volume of distribution. This way, the two effects share a similar

base relationship based on the first hidden layer which and individual differences between their effects on the different PK parameters can be learned based on the second set of hidden layers.

The second model (VWF:Ag on clearance) consisted of a single hidden layer of 12 neurons feeding into a single output neuron. Again the transformed softplus activation function was used. Inputs were normalized between roughly 0 and 1 by dividing model input by 350%. Global parameters were estimated for $Q$ and $V_2$. All parameters were constrained to be positive using a softplus activation function. We chose 12 neurons in all hidden layers as this allowed a sufficient level of complexity of the learned functions, while not being so large as to result in excessive overfitting (which could be likely when using 128 neurons for example). The number of neurons can potentially be optimized (by means of hyperparameter tuning), but we found the risks of overfitting to be already sufficiently managed when using 12 neurons. Bias parameters in the output layers were initialized to ones to initialize the model at reasonable estimates at the start of training.

## 2.3 Visualization of learned effects

Visualizations of learned functions were obtained by entering dummy input to each of the sub-networks. First, typical estimates for each of the PK parameters were obtained by dividing the prediction of each neural network to its prediction for the median covariate value (using typical clearance, $CL_{TV}$, as an example):

$$CL_{TV} = \frac{f_1(x_1)}{f_1(\text{Med}[x_1])} \cdot \frac{f_2(x_2)}{f_2(\text{Med}[x_2])} \tag{s14}$$

Here $f_1$ represents the subnetwork for the effect of weight (or fat-free mass), while $f_2$ represents the effect of VWF:Ag. We chose to use a value of 60 kg for fat-free mass, and 100% for VWF:Ag. Each model in the deep ensemble produces estimates of the typical value for the PK parameters. This way the prediction from each neural network are anchored to 1 at the median values of the covariates, similar to how covariates are implemented in non-linear mixed effects models. After calculation of the typical PK parameter estimates we can investigate the variance of these values over replicates to determine their uncertainty.

Predictions from each subnetwork divided by their prediction at the median covariate value can then be evaluated at any value of the covariate. We can thus visualize model predictions along the entire covariate space in order to obtain the visualizations.

## 2.4 Parameter initialization

Model parameters were randomly drawn from initial guess distributions at the start of optimization for each training replicate. Since the three optimization

algorithms share the same parameters ($\Theta = \{w, \Omega, \Sigma\}$), the same initial guess distributions were used.

- Neural network parameters $w$ were initialized using Xavier initialization: $w \sim \text{Uniform}(-x, x)$ where $x = \sqrt{(6/(in + out))}$ where $in$ and $out$ reference the number of input neurons and output neurons for that layer, respectively.
- Covariance matrix $\Omega$ is used in the prior distribution over the random effects $\eta \sim \mathcal{N}(\mathbf{0}, \Omega)$, and was decomposed into marginal standard deviations $\omega$ and correlation coefficient $\rho$. The following distributions were used for these parameters: $\omega \sim \text{Normal}(0.1, 0.025)$ truncated at [0, Inf] and $\rho \sim \text{Normal}(0, 0.1)$.
- Covariance matrix $\Sigma$ represents the estimates of residual error. The initial distribution for additive error was sampled from $\sigma \sim \text{Normal}(0.1, 0.025)$ truncated at [0, Inf]. The same distribution was used to sample the initial proportional error estimate whenever applicable.

The same initial guess distributions were used for the simulation and real-world experiments. Only additive error was used in the simulation experiment.

## 2.5 MCMC model

We first compared the accuracy of posterior distributions over the random effects $\eta$ obtained through MCMC and VI. We evaluated the different approaches in two setings: (1) the ground truth parameters used in the simulation were known (i.e. we only estimate posterior distributions over the random effects) and (2) only the typical PK parameters were known (i.e. also estimate posterior distributions over the population parameters $\Omega$ and $\Sigma$). For the MCMC model, we fit a single chain to the data. Since this problem was relatively simple, the model converged well and multiple chains were not required. Pseudo-code representing the probabilistic models are shown in listing 1.

The following hyper-priors were used in setting 2:
$S \sim \text{LogNormal}(-1.5, 1)$: marginal standard deviations of $\Omega$.
$\rho \sim \text{Beta}(2, 2)$: correlation coefficient in $\Omega$.
$\sigma \sim \text{LogNormal}(-3, 1)$: additive error.

```julia
using Turing

@model function model(zeta, omega, sigma, y) # setting (1).
    eta ~ MultivariateNormal(zeros(2), omega)
    z = zeta .* exp(eta) # individual estimates of PK parameters
    yhat = solve_ode(z)
    y ~ MultivariateNormal(yhat, sigma)
end

@model function model(zeta, y) # setting (2).
    sigma ~ LogNormal(-3, 1)
    S ~ LogNormal(-1.5, 1)
    rho ~ Beta(2, 2)
    C = [1 rho; rho 1]
    omega = S * C * S'
    eta ~ MultivariateNormal(zeros(2), omega)
    z = zeta .* exp(eta)
    yhat = solve_ode(z)
    y ~ MultivariateNormal(yhat, sigma)
end
```

**Listing 1:** Pseudo-code for MCMC models.

# Supplementary tables and figures

| VI algorithm | Wasserstein distance with respect to MCMC posterior $\times 10^{-3}$ Random effect, $\eta$ ($W_2 \pm$ SD) | Additive error, $\sigma$ ($W_1 \pm$ SD) | $\omega_1$ ($W_1 \pm$ SD) | $\omega_2$ ($W_1 \pm$ SD) | Correlation coefficient, $\rho$ ($W_1 \pm$ SD) |
|---|---|---|---|---|---|
| *Typical PK and population parameters known* | | | | | |
| Standard estimator | 9.0 ± 3.5 | - | - | - | - |
| Path derivative estimator | **5.5 ± 2.9** | - | - | - | - |
| *Typical PK parameters known* | | | | | |
| Standard estimator | 8.8 ± 3.3 | 5.0 ± 0.6 | 7.2 ± 3.1 | 10.9 ± 3.2 | 46.9 ± 23.9 |
| Path derivative estimator | **4.5 ± 2.3** | **0.6 ± 0.3** | **6.7 ± 3.5** | **7.2 ± 4.0** | **43.4 ± 14.3** |

Abbreviations: VI = Variational Inference, $W_2$ = 2-Wasserstein distance, $W_1$ = 1-Wasserstein distance, SD = standard deviation

**Supplementary Table 1:** *Accuracy of the variational posteriors compared to MCMC.*
Wasserstein distances were calculated with respect to multivariate normal distributions fit to the samples obtained through MCMC. For $\sigma$, $\omega_1$, and $\omega_2$, MCMC posteriors were represented by fitting a LogNormal distributions to the samples, while a SkewNormal distribution was fit to represent the posterior for $\rho$. Bold text represents the lowest Wasserstein distances.

| Method | Run time (minutes; median ± SD) | Final objective function value (median ± SD) | RMSE (IU/dL; median ± SD) | KL divergence of $\Omega$ (median ± SD) | MAE of $\omega_1 \pm$ SD | MAE of $\omega_2 \pm$ SD | MAE of $\sigma \pm$ SD (IU/dL) |
|---|---|---|---|---|---|---|---|
| One sample | 5.20 ± 0.41 | 298 ± 3.6[a] | 5.83 ± 0.68 | 0.0089 ± 0.022 | 0.014 ± 0.001 | 0.0029 ± 0.003 | 0.050 ± 0.042 |
| Three samples | 14.7 ± 2.6 | 309 ± 2.4[a] | 5.80 ± 0.59 | 0.011 ± 0.005 | 0.013 ± 0.008 | 0.0086 ± 0.002 | 0.23 ± 0.03 |

SD = standard deviation, RMSE = root mean squared error, KL = Kullback-Leibler, MAE = mean absolute error.
[a] = Based on stochastic estimates of the ELBO. Higher is better.

**Supplementary Table 2:** *Comparison of parameter estimates of VI objective based on the number of Monte Carlo samples.*
Results are shown for the VI models fit to the synthetic data experiment. Decreasing the number of Monte Carlo samples from three to one did not seem to affect parameter accuracy.

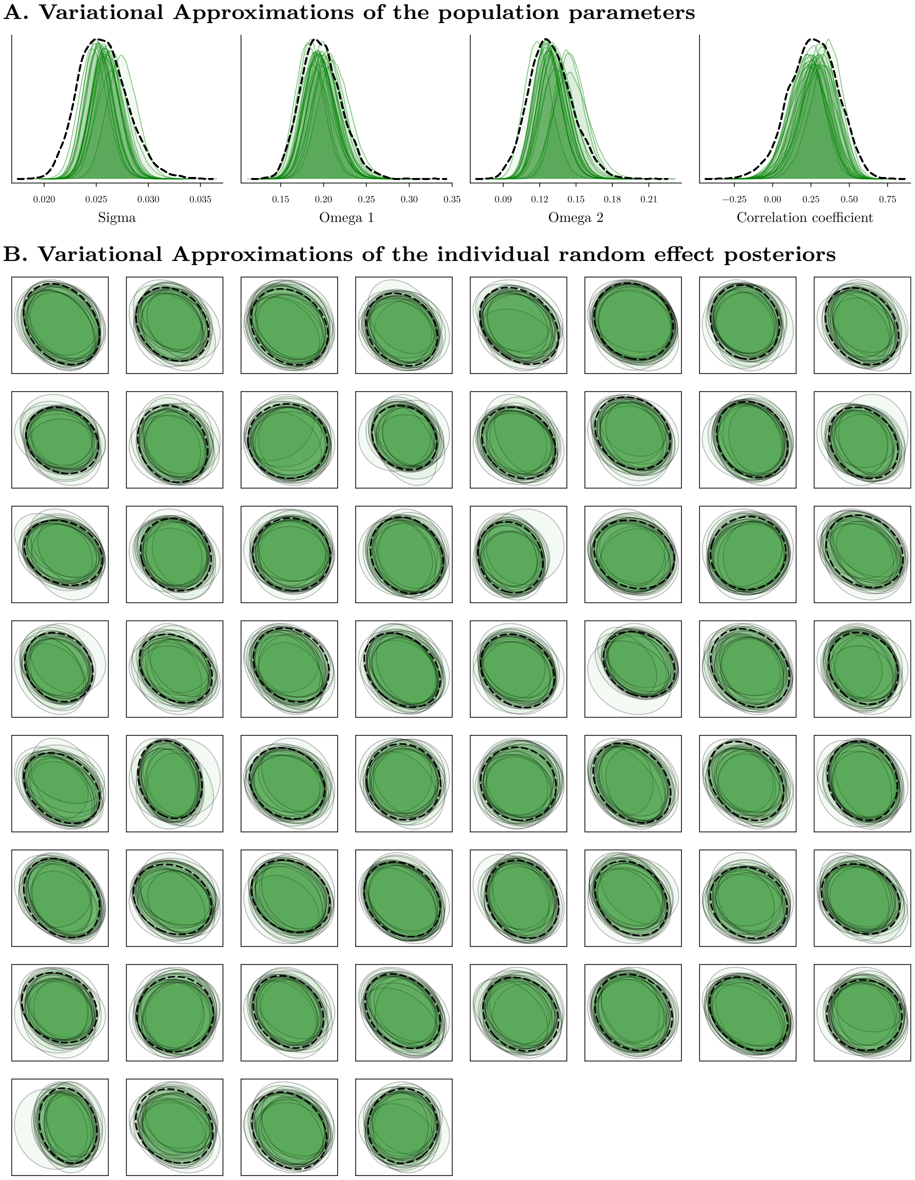


**Supplementary Figure 1:** *Posterior approximations using variational inference.*
Variational posteriors for the population parameters (A) as well as the individual random effect parameters (B) are shown. Black dashed lines represent posteriors obtained through MCMC. Results are shown for 20 replicates of the model fit using the path derivative gradient estimator.

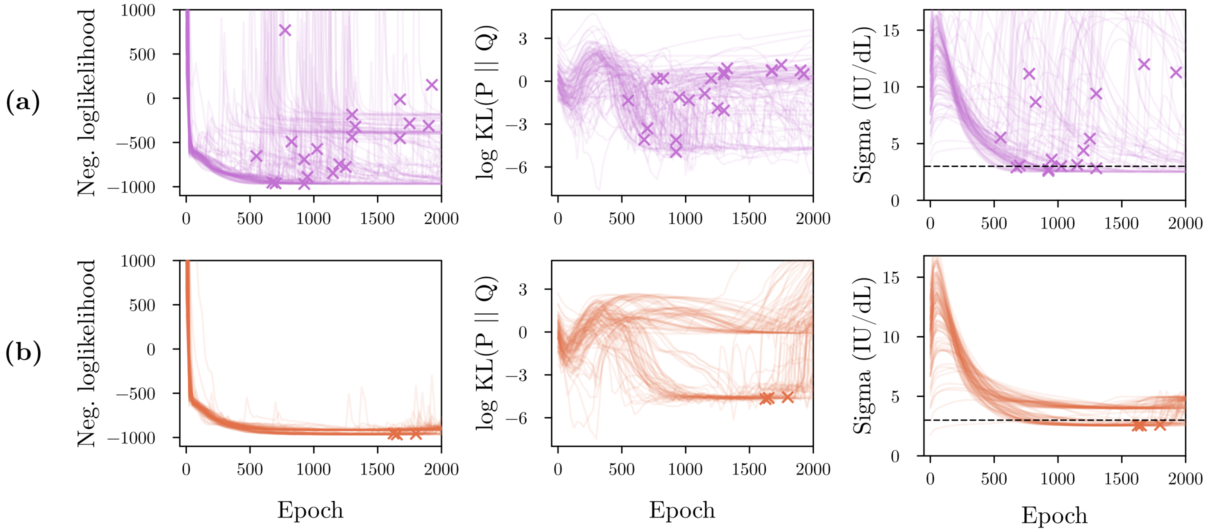


**Supplementary Figure 2:** *Objective function value and parameter accuracy for the FOCE objectives during training.*
The objective function value (left column), log KL divergence of the estimated random effect prior (centre column), and residual error estimate (right column) during training are shown for the FOCE based objectives. Results are shown for the FOCE objective according to equation s9 (a), equation s10 (b) using the reduced learning rate. Each line represents a single replicate fit to one of the data folds. Dashed line indicates the true value for sigma. Crosses indicate models that failed convergence. The formulation of the FOCE objective based on equation s9 (a) depicts lower stability during training and a higher fraction of models failing optimization.

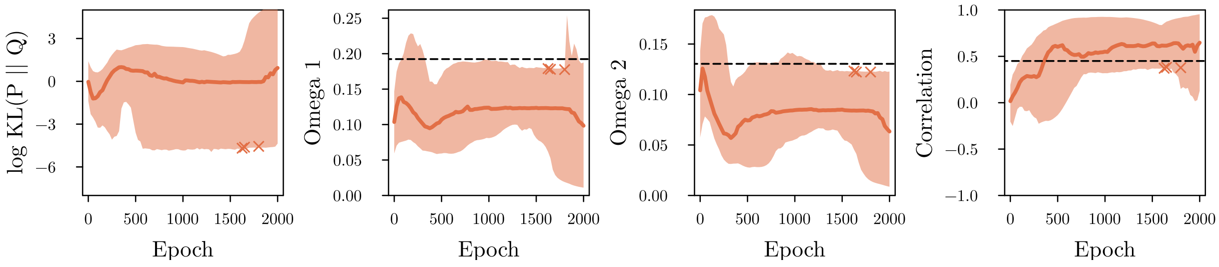


**(a)** **(b)** **(c)** **(d)**

**Supplementary Figure 3:** *Population parameter estimates during optimization using the FOCE objective.*
The log KL divergence of the estimated random effect prior (a), marginal standard deviation of $\omega_1$ (b) and $\omega_2$ (c), and their correlation coefficient (d) during training are shown. Results are shown for the FOCE objective according to equation s10 with reduced learning rate. Each line represents a single replicate along the data folds. Dashed lines indicate the true parameter value. Crosses represent early end of optimization due to errors. The model generally seems to underestimate the marginal variances of the true prior distribution.

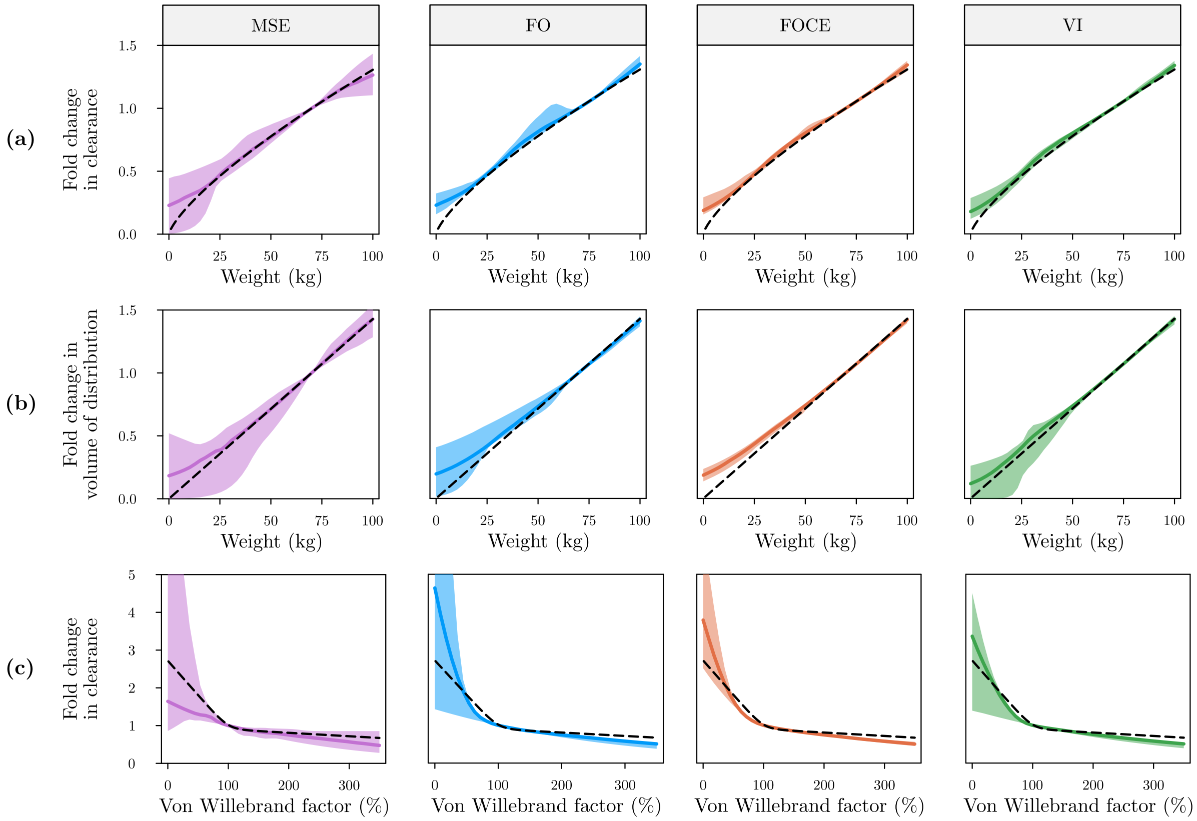


**Supplementary Figure 4:** *Learned covariate effects after training on the synthetic data set.*
Covariate effects for models fit using the FO (left column), FOCE (Eq. s10; center left column), VI (center right column), and mean squared error (right column) are shown. Learned functions are shown for the effect of weight on clearance (a), weight on volume of distribution (b) and von Willebrand factor on clearance (c). Median covariate effect (solid line) along with 95% confidence intervals are shown. Dashed black lines indicates the ground truth functions used in the simulation.

**1). Data set one (prophylactic setting)**

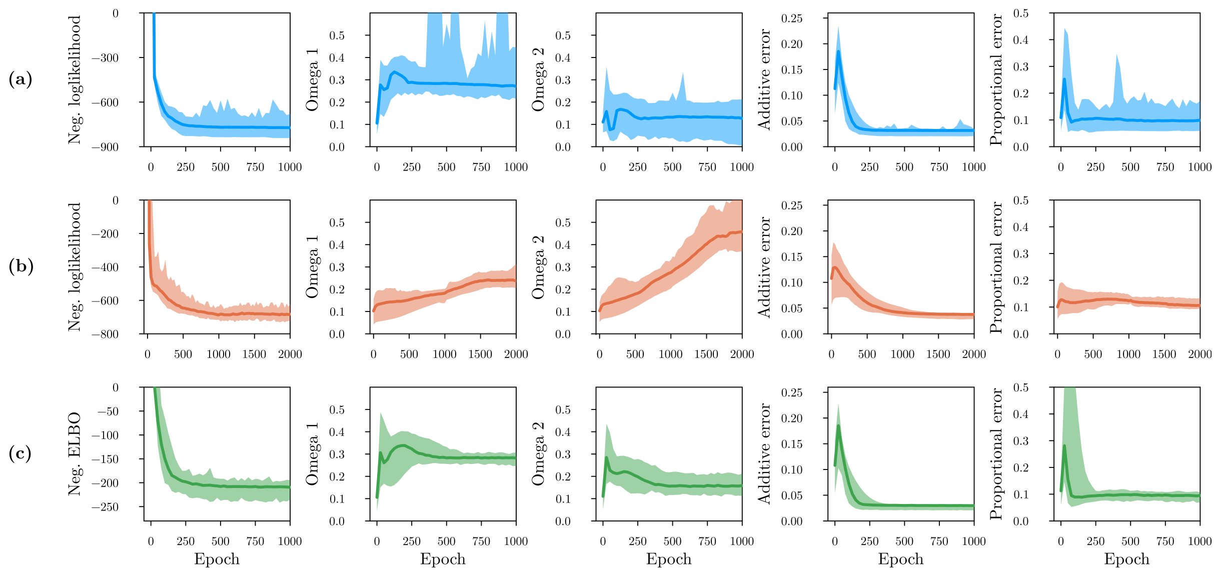


**2). Data set two (perioperative setting)**

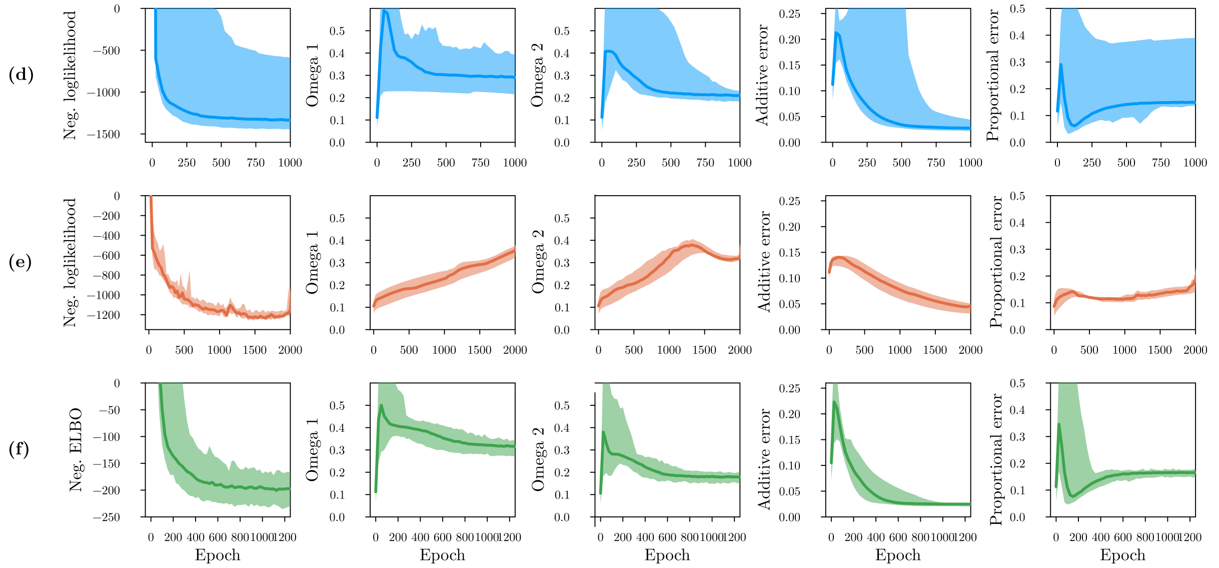


**Supplementary Figure 5:** *Parameter estimates during training on the real-world data sets.*
Parameter estimates during training are shown for the FO (a & d), FOCE (b & e), and VI (c & f) based objectives. Results are shown for data set one (a-c) and data set two (d-f). Median estimate (solid line) along with 95% confidence interval across replicates are shown.

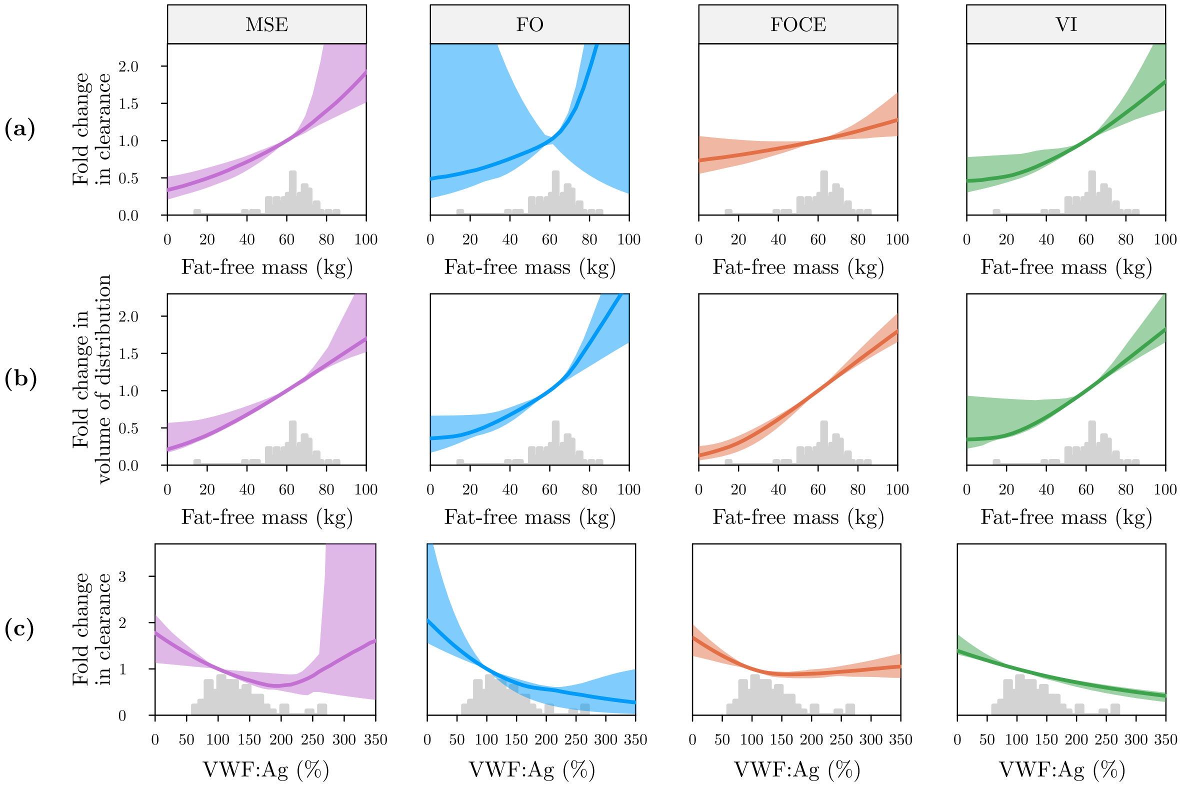


**Supplementary Figure 6:** *Learned functions after training on real-world data set two.*
Covariate effects for models fit using the MSE (left column), FO (centre left column), FOCE (centre right column), and VI (right column) are shown. Learned functions are shown for the effect of fat-free mass on clearance (a), fat-free mass on volume of distribution (b) and von Willebrand factor antigen (VWF:Ag) levels on clearance (c) at the end of training for data set two. Median covariate effect (solid line) along with 95% confidence intervals are shown. Grey histograms represent the corresponding covariate distributions.

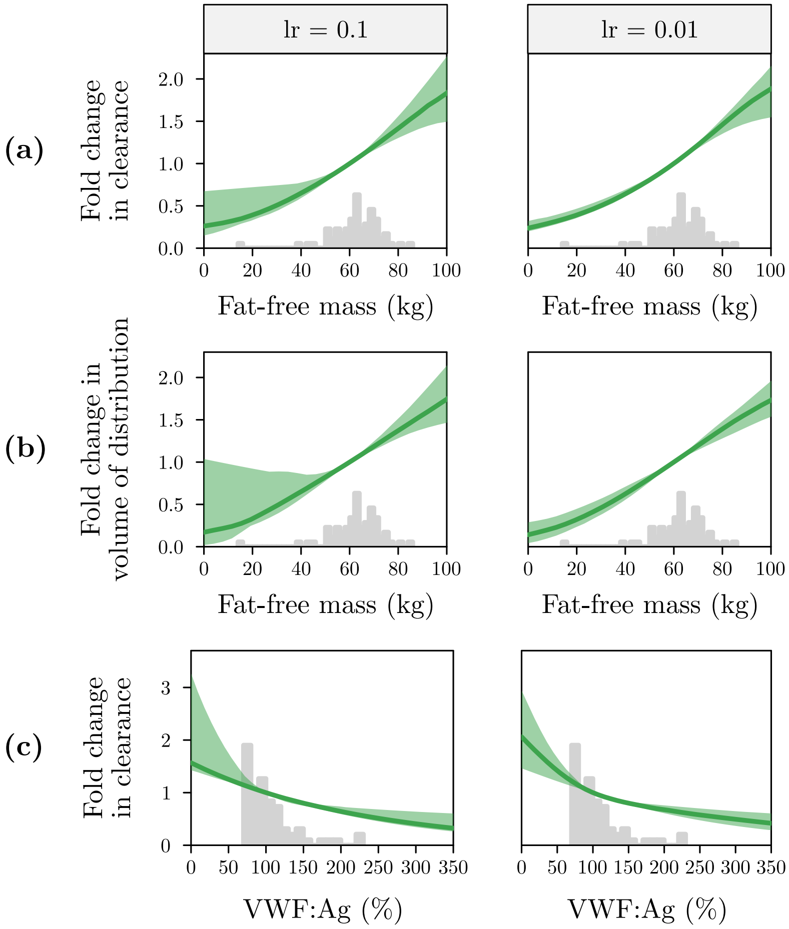


**Supplementary Figure 7:** *Decreasing the learning rate lowers uncertainty over learned effects for VI.*
Results are shown for the VI models trained using the regular learning rate (left column) and reduced learning rate (right column). Learned functions are shown for the effect of fat-free mass on clearance (a), fat-free mass on volume of distribution (b) and von Willebrand factor antigen levels on clearance (c) at the end of training for data set one. Median covariate effect (solid line) along with 95% confidence intervals are shown. Grey histograms represent the corresponding covariate distributions. lr = learning rate, VWF:Ag = von Willebrand factor antigen.
